# Diagnosis and Monitoring of Retinal Vasculitis by Widefield Swept Source OCT Angiography

**DOI:** 10.3390/diagnostics15243129

**Published:** 2025-12-09

**Authors:** Manish Harrigill, Matthew Nguyen, Jila Noori

**Affiliations:** 1 Dean McGee Eye Institute, University of Oklahoma Health Sciences Center, Oklahoma City, OK 73104, USA; 2 College of Medicine, The University of Oklahoma, Oklahoma City, OK 73104, USA; matthew-nguyen@ouhsc.edu

**Keywords:** retinal vasculitis, optical coherence tomography angiography, fluorescein angiography

## Abstract

**Objectives:** To evaluate the utility of widefield montage swept-source OCT angiography (SS-OCTA) in detecting and monitoring retinal vasculitis beyond the posterior pole. **Methods:** Prospective case series. Patients with clinically diagnosed retinal vasculitis imaged with a same-day widefield SS-OCTA montage and ultra-widefield fluorescein angiography (FA) at 2 or more visits. Five overlapping 12 × 12 mm SS-OCTA scans were acquired to provide imaging of the posterior pole and each quadrant of the near periphery. A color retinal thickness map was superimposed on each 12 × 12 mm en-face flow scan with a customized segmentation to demonstrate perivascular retinal thickening. A composite “montage” image was then created by combining the scans to allow for analysis of the macula and near periphery. Findings were then correlated with the same-day FA, the current “gold standard” diagnostic tool for retinal vasculitis, to assess diagnostic efficacy. **Results:** SS-OCTA demonstrated perivascular thickening in both the posterior pole and peripheral retina in 30 eyes of 16 patients and was found to be an effective diagnostic tool with good correlation to findings on fluorescein angiography for monitoring retinal vasculitis over time. **Conclusions:** The widefield SS-OCTA montage expands the visualization of retinal vasculitis into the near periphery, providing a noninvasive tool that may complement FA in the diagnosis and monitoring of retinal vasculitis.

## 1. Introduction

Retinal vasculitis is a sight-threatening complication of both ocular inflammatory/infectious diseases, such as herpetic retinitis, birdshot chorioretinopathy, and pars planitis, as well as systemic infectious and autoimmune conditions, including syphilis, Lyme disease, sarcoidosis, lupus, and Behçet disease. Histopathologic studies consistently demonstrate that the underlying process is best described as perivascular infiltration of inflammatory cells, rather than primary vasculitis of the vessel wall [1,2,3,4]. This observation has important diagnostic implications: since the pathophysiologic hallmark of retinal vasculitis is perivascular inflammation and edema, imaging modalities capable of detecting perivascular thickening may provide a direct and clinically meaningful measure of disease activity.

Fluorescein angiography (FA) has long been the gold standard for diagnosis and monitoring, highlighting vascular leakage and staining, as well as secondary findings such as macular edema, vascular occlusion, optic disk edema, and neovascularization [5,6]. Despite its diagnostic utility, FA is invasive, time-intensive, and associated with both common adverse effects (e.g., nausea, vomiting) and rare but severe complications such as anaphylaxis [7].

Optical coherence tomography angiography (OCTA) is a noninvasive modality that provides high-resolution, depth-resolved en-face visualization of the retinal vasculature by detecting motion contrast from flowing erythrocytes [8]. OCTA has significantly advanced the study of retinal vascular disease, offering precise mapping of ischemia and microvascular remodeling. In retinal vasculitis, OCTA is sensitive for detecting nonperfusion; however, it has historically been limited in demonstrating active inflammation because leakage is not directly visualized [9,10,11].

Recent studies, however, have bridged this gap by showing that OCTA can indirectly detect inflammatory activity through structural correlates. Noori et al. demonstrated that swept-source OCTA (SS-OCTA) can identify perivascular retinal thickening that correlates closely with areas of FA leakage in the posterior pole, providing a surrogate marker of inflammation and a tool for monitoring treatment response [12]. Similarly, widefield OCTA has shown potential to capture peripheral areas of nonperfusion; however, its role in diagnosing active vasculitis remains incompletely defined [13,14].

The purpose of the present study is to extend these findings by evaluating the use of the widefield SS-OCTA montage, created from five 12 × 12 mm scans, in diagnosing and monitoring retinal vasculitis. To our knowledge, no prior study has systematically applied widefield OCTA montage to assess both posterior pole and peripheral involvement over time. We demonstrate that this technique provides a novel, effective, and noninvasive tool for the detection and longitudinal assessment of disease activity.

## 2. Materials and Methods

### 2.1. Study Design and Patient Selection

This prospective case series was conducted in compliance with the Declaration of Helsinki and the Health Insurance Portability and Accountability Act of 1996, and received approval from the Institutional Review Board of the University of Oklahoma College of Medicine. All participants gave written, study-specific informed consent. Eligible patients were identified from the uveitis clinic if they had fluorescein angiography (FA) evidence of retinal vasculitis and consented to same-day widefield SS-OCTA montage imaging and FA. Inclusion required both imaging modalities to be performed during two or more separate visits between May 2024 and July 2025. For each participant, clinical data—including age, underlying diagnosis, current and past treatments, and FA interpretations from each visit—were collected. The severity of anterior chamber and vitreous inflammation was assessed at each visit. Additional findings, such as chorioretinal lesions, perivascular exudates, intraretinal hemorrhages, optic nerve edema, macular edema, and epiretinal membranes, were also documented.

### 2.2. Fluorescein Angiography

All FA imaging was performed using an ultra-widefield device (Optos, Optos PLC, Dunfermline, UK). Available FA frames from each visit were reviewed in full, and representative early-phase images (approximately 1–2 min) and late-phase images (4–10 min) were selected for comparison with OCTA montage images. FA served as the reference standard for the presence of perivascular leakage and staining.

### 2.3. OCTA Image Acquisition and Segmentation

**Figure 1:**
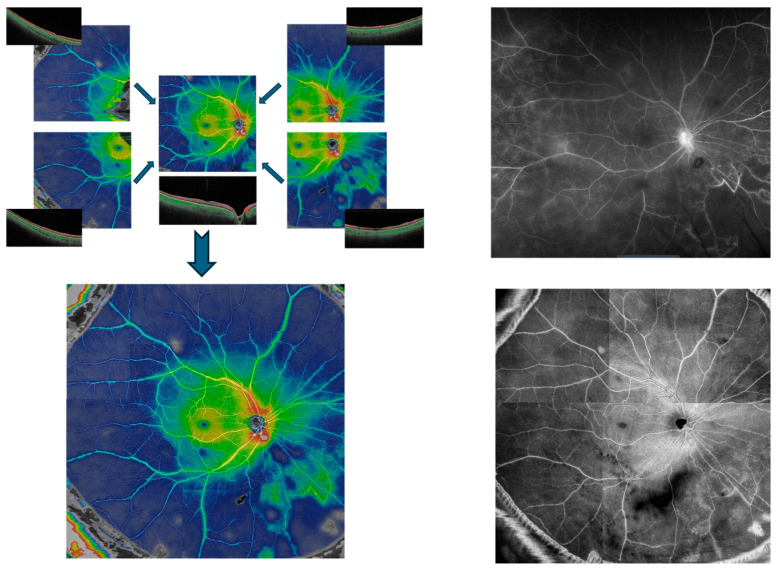
Creation of a Widefield SS-OCTA montage. Five individual 12 × 12 mm SS-OCTA scans of macula and peripheral quadrants (**upper left**) are manually aligned to compose widefield montage image (**lower left**), demonstrating active vasculitis in the right eye of a patient with Behcet’s disease. The montage en-face scan (**lower right**) and a late phase image of the same-day widefield fluorescein angiography (**upper right**) are shown for comparison.

SS-OCTA imaging was performed with the PLEX Elite 9000 (Carl Zeiss Meditec, Jena, Germany). For each patient eye, five 12 × 12 mm scans were obtained—one centered on the fovea and one from each quadrant of the near periphery (superonasal, superotemporal, inferonasal, and inferotemporal)—which were then manually aligned to create a widefield montage (Figure 1).

**Figure 2:**
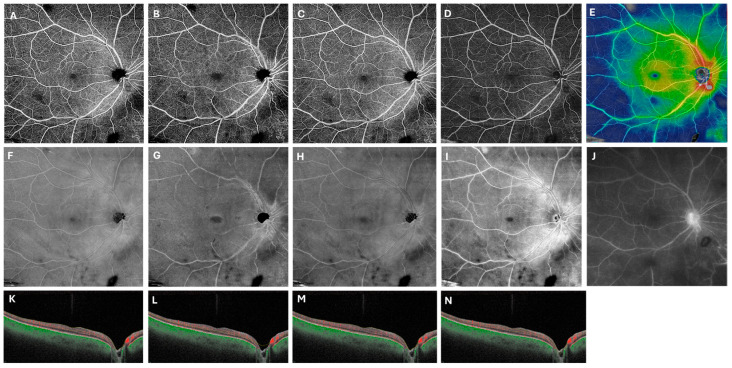
Customized segmentation enhances visualization of retinal vasculitis on swept-source OCT angiography. In the right eye of a patient with Behcet panuveitis, the 12 × 12 mm default superficial (**A**,**F**,**K**), deep (**B**,**G**,**L**), and whole retina scans (**C**,**H**,**M**) are compared with the customized scans (**D**,**I**,**N**). The first row displays flow en-face scans, while image (**E**) presents a composite flow en-face scan overlaid with a color thickness map. Fluorescein angiography (**F**) is provided for reference. The second row (**F**–**I**) shows structural en-face scans, with the customized slab more closely resembling the vessel patterns seen on FA (**J**). The last row includes B-scan with red segmentation lines for each slab.

A customized retinal segmentation identical to that described by Noori et al. was applied, extending from 49 µm to 149 µm beneath the internal limiting membrane [12]. This segmentation was chosen because it most closely approximates the contrast of FA images, facilitating visualization of the retinal vasculature against a dark background. In addition, this segmentation reduces artifacts from nerve fiber layer hyperreflectivity and encompasses both the superficial and deep capillary plexuses. Figure 2 illustrates this process in the representative patient from Figure 1 with Behcet’s disease, showing late-phase FA with vessel staining alongside the corresponding OCTA flow en-face, structural en-face, and composed color thickness map images, demonstrating perivascular thickening in hot colors.

### 2.4. Image Processing and Montage Creation

Automated retinal thickness color maps were superimposed on each flow en-face scan to highlight areas of perivascular retinal thickening. Cooler colors (green and blue) correspond to thinner retina, while warmer colors (yellow, orange, and red) indicate thicker retina. Modified flow en-face scans with overlaid thickness maps were generated for all five fields and manually aligned based on retinal vascular landmarks to create a widefield composite “montage” image of the posterior pole and near periphery.

In addition, structural en-face scans were analyzed as ancillary data. These scans used the same customized segmentation boundaries but were not overlaid with a color thickness map. Structural en-face montage images were created from the same five scan fields using the same alignment process.

### 2.5. Image Analysis

Composite flow/thickness montage and structural en-face montage images were compared with a corresponding FA image captured from 4 to 10 min at each visit. Since all patients had multiple visits, serial montage OCTA images were also analyzed over time to assess correlation with FA leakage and to evaluate changes in perivascular thickening with treatment response.

## 3. Results

### 3.1. Patient Characteristics

A total of 30 eyes from 16 patients with a diagnosis of retinal vasculitis were included.

The mean age was 53.7 (24–71), with 4 male and 12 female patients. The underlying diagnoses included Birdshot Chorioretinopathy, Behçet’s Disease, HLA B27-associated uveitis, sarcoidosis, syphilis, and sympathetic ophthalmia. A summary of patient demographics, systemic diagnoses, ocular findings, treatments, and FA findings are provided in Table 1.

### 3.2. Correlation of OCTA Montage with FA

**Figure 3:**
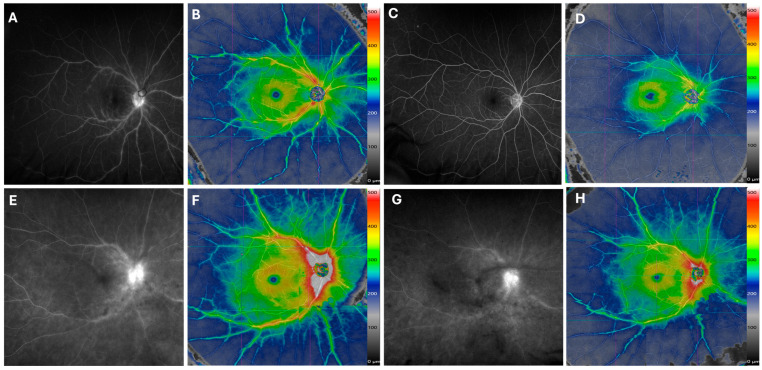
Improvement of Retinal Vasculitis Following Treatment. The late-phase fluorescein angiography (FA) of a patient with idiopathic retinal vasculitis reveals vascular staining (**A**) and perivascular retinal thickening in hot colors on the same-day widefield SS-OCTA montage (**B**). After 8 months of treatment with mycophenolate and adalimumab, both FA (**C**) and widefield SS-OCTA (**D**) show marked improvement. Similarly, another patient with undifferentiated intermediate uveitis and retinal vasculitis demonstrates reduced vascular leakage and staining on both FA and widefield SS-OCTA after 5 months of Humira treatment in the right eye (**E**–**H**). A color scale bar is provided next to montage OCTA scans to indicate the range of change in perivascular retinal thickness in areas of improvement. The color map illustrates relative thickening; its colors do not indicate absolute thickness (µm), and there are no established cutoff values for retinal vasculitis activity.

**Figure 4:**
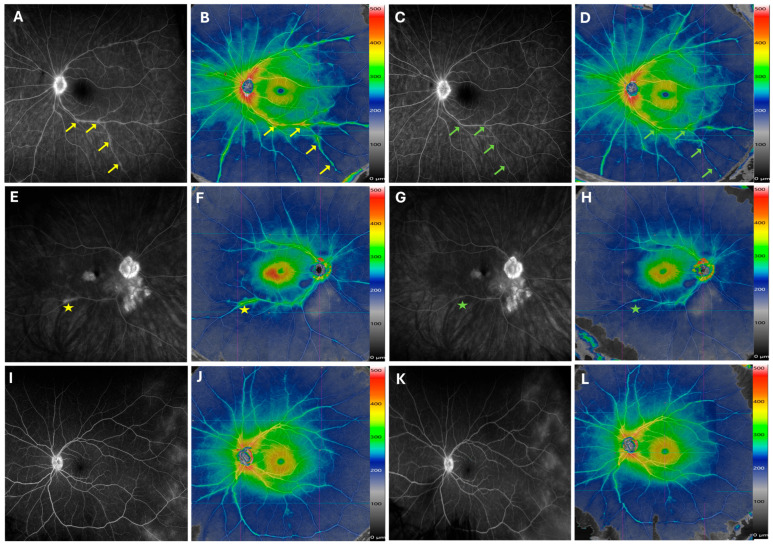
Longitudinal Changes in Retinal Vasculitis During Therapy Escalation. Late-phase fluorescein angiography (FA) (**A**) and widefield SS-OCTA montage (**B**) from a patient with newly diagnosed birdshot chorioretinopathy show improvement in phlebitis, seen on FA (**C**) and widefield SS-OCTA (**D**), after 6 months of methotrexate treatment. The improvement is especially notable at the inferior arcade venule. The improved segments along the venular branches are marked by arrows. In the right eye of another patient with birdshot chorioretinopathy, both FA and SS-OCTA demonstrate reduced inferior arcade venule staining and central macular edema (CME) 8 months after intravitreal fluocinolone implant injection (**E**–**H**). The improved area is marked by a star sign. For a patient with idiopathic uveitis, vascular leakage in the temporal retina is visible on FA but not captured on widefield montage SS-OCTA at either baseline or the 6-month follow-up visit (**I**–**L**).

There was good overall correlation between areas of perivascular retinal thickening on OCTA color thickness maps and regions of vascular leakage or staining on FA. This was consistently observed across both posterior pole and near peripheral montage fields. Representative cases are presented in Figure 3 and Figure 4, which demonstrate close spatial agreement between OCTA color thickness maps and FA images.

The color thickness maps were the primary modality used for OCTA–FA comparison, though structural montage scans were also analyzed as ancillary data to confirm anatomic correlates.

### 3.3. Longitudinal Assessment

Serial SS-OCTA montages were available for all patients. In many cases, OCTA effectively captured changes in disease activity over time. Eyes that showed clinical improvement and reduced leakage on FA demonstrated corresponding decreases in perivascular retinal thickening on OCTA, while eyes with worsening or recurrent disease showed progressive thickening. Examples of improvement over time are highlighted in Figure 3.

## 4. Discussion

Since its clinical adoption, OCTA has transformed the study of retinal vascular disease by enabling noninvasive, depth-resolved visualization of the retinal microvasculature and perfusion dynamics. This capability has broadened from macular disorders to widefield applications in infectious and inflammatory diseases.

Within retinal vasculitis, OCTA has consistently been shown to demonstrate areas of retinal nonperfusion but not to capture active inflammation [13,14,15]. Noori et al., however, demonstrated that SS-OCTA can reveal perivascular retinal thickening that corresponds to vascular leakage and staining seen on FA, introducing a structural OCTA biomarker that tracks disease activity and treatment response [12]. Our work builds directly on this foundation and extends it spatially using a five-field 12 × 12 mm montage to capture both posterior pole and near periphery over time.

We found strong qualitative agreement between OCTA color thickness maps and FA leakage/staining across montage fields, and we show that serial montage imaging sensitively reflects improvement or worsening in disease course, which are practical advantages for longitudinal care. These findings complement earlier reports comparing OCTA and FA in retinal vasculitis and intermediate uveitis by illustrating how perivascular thickening plus widefield coverage can enhance assessment of disease activity [13,14].

Histopathologic work has long shown perivascular inflammatory infiltration as a defining feature of retinal vasculitis; the present method’s emphasis on perivascular thickness makes biological sense and provides an intuitive, color-encoded surrogate for active inflammation that clinicians can track. Together with the high-resolution vascular detail intrinsic to OCTA, the superimposed thickness map creates a visually efficient diagnostic composite for both detection and monitoring. A recent systematic review of OCT/OCTA in retinal vasculitis highlights this evolving role and cites Noori et al. as a key step toward OCTA-based monitoring; our findings extend that line of evidence to widefield montage and longitudinal application [15].

This study has several limitations. The field of view of the montage scan does not capture the far periphery, and our five 12 × 12 mm montage effectively images retinal Zones 1–2, but Zone 3 remains outside the current capture area, potentially missing very peripheral pathology (Figure 4I–L).

Another limitation is imaging artifacts. OCTA is vulnerable to motion, projection, and segmentation artifacts, which led to the exclusion of several visits due to uninterpretable scans. This challenge reflects real-world constraints reported across devices and cohorts [16,17]. Segmentation variability and other structural confounders—such as traction, edema, and epiretinal membrane—can also produce warm colors on the map. In these cases, correlation with B scans is necessary to properly interpret findings, as these artifacts may mimic perivascular retinal thickness secondary to retinal vasculitis [12].

Technical factors inherent to the widefield montage technique can introduce subtle variability. Each 12 × 12 mm OCTA scan generates an independent retinal thickness map based on its own signal strength and local segmentation. Assembling multiple color-coded images into a montage inherently introduces inter-field variability and may influence measured retinal thickness in peripheral regions. Additionally, manual alignment of overlapping scans can create minor stitching artifacts or geometric misregistration, further affecting the accuracy of composite flow and thickness maps.

This variability may also affect the accuracy of comparison between a montage system and an ultra-widefield FA system, which provides a single capture, uniformly illuminated imaging without the geometrical variability introduced by multiple acquisitions. This may limit the reliability of comparing pre- and post-treatment montage scans, as small changes in variables such as vitreous haze, fixation, and signal strength may alter visit-to-visit scan quality independent of the true structural changes secondary to retinal vasculitis.

Although qualitative agreement between OCTA and FA was visually strong, our analysis remains descriptive, as this report lacks quantitative measures. Nevertheless, the color-coded thickness scale adjacent to the SS-OCTA montage provides a coarse estimate of quantitative change over time (Figure 3 and Figure 4). The color maps illustrate relative changes in retinal thickness but are not validated quantitative biomarkers with defined numerical thresholds. In montage configurations, they do not support pixel-wise thickness export, which limits reproducible quantitative analysis. Meanwhile, we are developing a new biomarker independent of the thickness map that will require validation in a larger cohort stratified by disease phase or treatment status.

Future work should focus on prospective, blinded studies with predefined grading protocols to quantify sensitivity, specificity, and reproducibility of OCTA montage (flow scan with thickness overlays) relative to FA. Furthermore, establishing a reproducible quantitative biomarker that specifically identifies increased perivascular retinal thickness due to retinal vasculitis—while excluding other etiologies—would add a robust quantitative measure to validate this method for detecting and monitoring retinal vasculitis.

## 5. Conclusions

Widefield SS-OCTA montage with integrated color thickness maps is a novel, practical, and noninvasive approach for diagnosing and monitoring retinal vasculitis beyond the posterior pole. Building on the perivascular-thickening biomarker proposed by Noori et al., our longitudinal case series shows strong correspondence with FA and clear tracking of disease trajectory over time, supporting OCTA montage as a clinically useful adjunct, and in selected scenarios, it is a potential alternative to FA for ongoing management of retinal vasculitis.

## Figures and Tables

**Table 1:** Patient characteristics and clinical findings.

Patient No	Patient Gender	Patient Age	R/L Eye	Underlying Diagnosis	Uveitis Type	Macular Edema	Epiretinal Membrane	Treatment	Leakage on FA	Staining on FA	Zones on FA	Disease Activity Trend Based on FA/OCTA Findings	Visits with the Same-Day FA/OCTA	Best Corrected Visual Acuity (BCVA) at Each Visit	Follow-Up Interval
1	Female	24	OU	Idiopathic	Anterior/ Intermediate	No	No	Adalimumab	Yes	No	Zone 3 OU	Improved OU	2	Visit 1: 20/20 OU Visit 2: 20/20 OU	6 months
2	Female	60	OU	Idiopathic	Anterior/ Intermediate	No	Yes	Methotrexate	Yes	Yes	Zone 1–3 OU	Improved OU	2	Visit 1: 20/30 OD, 20/40 OS Visit 2:20/50 OD, 20/30 OS	5 months
3	Female	71	OU	Birdshot Chorioretinopathy	Panuveitis	No	Yes	OU Fluocinolone Acetonide 0.18 mg injection	No	Yes	Zone 1, 2 OU	Improved OU	3	Visit 1: 20/25 OD, 20/60 OS Visit 2: 20/20 OD, 20/25 OS Visit 3:20/25 OD, 20/40 OS	5 months, 6 months
4	Male	38	OD	Behcet’s	Panuveitis	No	No	Methotrexate, Adalimumab	Yes	Yes	Zone 1–3 OD	Worsened at visit 2, improved at visit 3	3	Visit 1: 20/25 OD Visit 2: 20/40 OD Visit 3: 20/40 OD	4 months, 4 months
5	Female	69	OU	Birdshot Chorioretinopathy	Panuveitis	Yes	No	Fluocinolone Acetonide 0.18 mg injection OD	No	Yes	Zone 1, 2 OU	Improved OD, Stable OS	2	Visit 1: 20/25 OD, 20/25 OS Visit 2: 20/20 OD, 20/20 OS	8 months
6	Female	45	OU	Idiopathic	Anterior/ Intermediate	No	No	Methotrexate	Yes	No	Zone 3 OU	Improved OU	2	Visit 1: 20/20 OD, 20/20 OS Visit 2: 20/20 OD, 20/20 OS	5 months
7	Male	44	OU	Birdshot Chorioretinopathy	Panuveitis	No	Yes	Methotrexate, Adalimumab	No	Yes	Zone 1–2 OU	Improved OU	2	Visit 1: 20/20 OD, 20/20 OS Visit 2: 20/20 OD, 20/20 OS	6 months
8	Female	70	OU	HLA B27 associated Uveitis	Intermediate	Yes	Yes	Mycophenolate	Yes	No	Zone 1–3 OU	Worsened OD, Stable OS	2	Visit 1: 20/20 OD, 20/20 OS Visit 2: 20/20 OD, 20/20 OS	8 months
9	Female	39	OU	Birdshot Chorioretinopathy	Panuveitis	No	No	Methotrexate, Adalimumab	Yes	Yes	Zone 1, 2 OU	Worsened OU	3	Visit 1: 20/80 OD, 20/25 OS Visit 2: 20/200 OD, 20/20 OS Visit 3: 20/100 OD, 20/20 OS	7 months, 6 months
10	Female	55	OU	Sarcoid	Panuveitis	No	Yes	Methotrexate, Adalimumab	Yes	Yes	Zone 2–3 OU	Worsened OD at visit 2 then improved. Stable OS	3	Visit 1: 20/20 OD, 20/25 OS Visit 2:20/20 OD, 20/20 OS Visit 3: 20/20 OD, 20/20 OS	5 months, 5 months
11	Female	35	OU	Syphilis	Panuveitis	No	Yes	Oral Steroid	Yes	Yes	Zone 2, 3 OU	Improved OU	2	Visit 1: 20/40 OD, 20/50 OS Visit 2: 20/20 OD, 20/20 OS	7 months
12	Female	60	OU	Birdshot Chorioretinopathy	Panuveitis	No	Yes	Methotrexate, Adalimumab	Yes	Yes	Zone 1, 2 OU	Improved OU	2	Visit 1: 20/20 OD, 20/20 OS Visit 2: 20/20 OD, 20/20 OS	9 months
13	Female	55	OU	Idiopathic	Posterior	No	Yes	Methotrexate, Adalimumab,	Yes	Yes	Zone 1–3 OU	Worsened OU	4	Visit 1: 20/20 OD, 20/25 OS Visit 2:20/25 OD, 20/20 OS Visit 3:20/30 OD, 20/25 OS Visit 4:20/25 OD, 20/25 OS	6 months, 6 months, 5 months
14	Male	57	OD	Sympathetic Ophthalmia	Panuveitis	Yes	Yes	Mycophenolate	Yes	no	Zone 1–3	Improved	2	Visit 1: 20/25 OD Visit 2: 20/30 OD	14 months
15	Female	69	OU	Birdshot Chorioretinopathy	Panuveitis	No	Yes	Adalimumab Mycophenolate	No	Yes	Zone 1, 2 OU	Improved OD, Worsened OS	3	Visit 1: 20/40 OD, 20/25 OS Visit 2: 20/40 OD, 20/25 OS Visit 3: 20/30 OD, 20/25 OS	8 months, 6 months
16	Male	68	OU	Sarcoidosis	Isolated Retinal Vasculitis	No	Yes	Mycophenolate	Yes	No	Zone 1–3 OU	Improved OU	2	Visit 1: 20/25 OD, 20/40 OS Visit 2: 20/30 OD, 20/30 OS	9 months

## Data Availability

The data supporting the findings of this study are not publicly available due to patient privacy and ethical restrictions. De-identified data may be made available from the corresponding author upon reasonable request and with appropriate institutional approvals.

## Abbreviations

The following abbreviations are used in this manuscript:

SS-OCTA	Swept-Source Optical Coherence Angiography Tomography
FA	Fluorescein Angiogram/Angiography
OCT	Optical Coherence Tomography
OCTA	Optical Coherence Tomography Angiography
